# Magnetic-Guided Capsule Endoscopy in the Diagnosis of Gastrointestinal Diseases in Minors

**DOI:** 10.1155/2018/4248792

**Published:** 2018-09-18

**Authors:** Yuting Qian, Tingting Bai, Juanjuan Li, Yi Zang, Tong Li, Mingping Xie, Qi Wang, Lifu Wang, Ruizhe Shen

**Affiliations:** Department of Gastroenterology, Ruijin Hospital Affiliated to Shanghai Jiaotong University School of Medicine, 197 Second Ruijin Road, Shanghai 200025, China

## Abstract

**Objective:**

This study aimed at investigating the clinical value of magnetic-guided capsule endoscopy (MGCE) in the diagnosis of gastrointestinal diseases in minors.

**Methods:**

Eighty-four minor patients hospitalized in the pediatric department at Ruijin Hospital between June 2015 and January 2018 were enrolled for this study. Following bowel preparation, all patients underwent MGCE. The feasibility, safety, diagnostic yield, and sensitivity of MGCE were analyzed. Patients were followed up for more than 2 weeks.

**Results:**

The main indications for MGCE in minors were Crohn's disease, gastrointestinal bleeding, and abdominal pain. The main causes of gastric disease were gastric inflammatory hyperplasia, exudative gastritis, and polyps. The most common small bowel diseases in minors were Crohn's disease, Henoch-Schonlein purpura, and polyps. The diagnostic yield in the stomach and small intestine was 13.1% and 28.6%, respectively, and the sensitivity was 100% and 96.0%, respectively. No adverse events occurred.

**Conclusion:**

MGCE is a safe, effective, and well-tolerated procedure with good sensitivity and has a potential clinic value for the diagnosis of gastrointestinal diseases in minors.

## 1. Introduction

Minor is a special group of patients, whose symptoms and diseases are often different from those of adults, especially for gastrointestinal diseases. In the past 30 years, esophagogastroduodenoscopy (EGD) has been considered as the gold standard for gastric disease investigation but the discomfort limits its use in minors. The appearance of capsule endoscopy (CE) has become a milestone in small bowel examination [1–3]. In 2009, indications of CE have been broadened to patients more than 2 years old by the United States Food and Drug Administration (FDA) and CE is proven safe to pediatrics [4–8]. With the continuous innovation of CE technology, magnetic-guided capsule endoscopy (MGCE) shows its advantages in gastric disease investigation [9–12]. However, there is a lack of the clinical value of magnetic-guided capsule endoscopy in pediatric gastrointestinal diseases. Until now, the decision of the clinical value of MGCE is based on adult research and empirical data based on CE [5, 13, 14]. Therefore, this prospective study aims at investigating the diagnostic value of magnetic-guided capsule endoscopy for minors' gastrointestinal diseases.

## 2. Materials and Methods

### 2.1. Study Cohorts

This study recruited patients from 6 to 18 years old hospitalized in the pediatric department from June 2015 to January 2018 at Ruijin Hospital Affiliated to Shanghai Jiaotong University, and the study was approved by the Ruijin Hospital Ethics Committee. Inclusion criteria were IBDU (inflammatory dowel disease unclassified), suspected Crohn's disease, hematemesis, hematochezia, melena, anemia, abdominal pain, diarrhea, abdominal distension, elevated tumor markers, constipation, and dyspepsia. Patients were divided into five groups by complains. The main exclusion criteria were metal implants (cardiac pacemaker, metal valves, metal prosthesis, etc.), impairment of gastrointestinal movement, suspected or diagnosed gastrointestinal obstruction, and history of abdominal surgery. All patients and their guardians were informed of the related process and potential risks during MGCE examination, and all signed consents.

### 2.2. Magnetic-Guided Capsule Endoscopy System

The NaviCam™ magnetic-guided capsule endoscopy system (Shanghai ANKON Medical Technology Co. Ltd.) was applied in this study. The components included capsule robot, magnetic-guided capsule endoscopy examination bed, translation and rotary table, magnet, console, portable recorder, capsule locator, and ESNavi software. The capsule robot was a capsule-like equipment with the size of 12 mm × 28 mm and took 2 frames per second. The observation view was 140 ± 10°, the working temperature was 20–40°C, and the working time was >8 h. The captured data of the capsule can be instantly transmitted by the data line to the operating table for real-time observation. The activity of the capsule was controlled by the C-arm magnetic field system.

### 2.3. Preparations

CTE or MRE examination was performed before MGCE examination to exclude intestinal obstruction. Subjects were required to carry out low-residue diet 3 days before the examination. At 8 pm the day before MGCE examination, patients drank polyethylene glycol electrolyte powder (Hengkangzhengqing, Jiangxi Hengkang Pharmaceutical Co. Ltd.) for intestinal cleaning. Patients younger than 10 years old or less than 40 kg took 25 ml/kg laxative, and patients older than 10 years and more than 40 kg took 2000 ml of laxative. On the day of examination, the subjects took 200–300 ml of water at 6 am. 10 ml of simethicone (Bo Xi, Berlin-Chemie AG) was taken 60 min before the examination. All metal belongings were removed (keys, metal dentures, mobile phones, watches, magnetic cards, etc.). Patients were demanded to take in 100 ml to 200 ml before MGCE examination. During the inspection process, if the vision was not clear, patients were asked to continue drinking water until the field of view was satisfied.

### 2.4. Procedures

**Figure 1:**
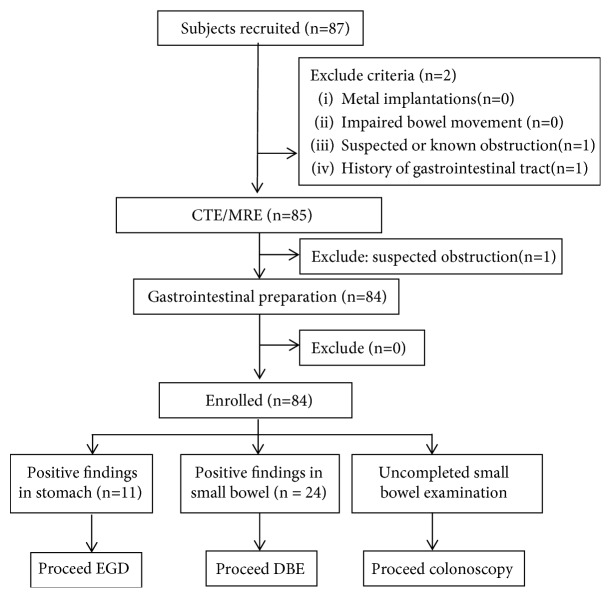
Flow chart of the study. EGD: esophagogastroduodenoscopy; DBE: double-balloon enteroscopy.

The patients put on the portable recorder and lied on the operating bed, then swallowed the capsule robot with 5 ml water through a suction tube. The physician blinded to CTE/MRE result carried out real-time monitoring. During the gastric examination, patients changed positions as ordered for better gastric observation. After finishing gastric investigation, the patients kept walking around and the capsules' location was evaluated 2 h later. Capsule retention in the stomach over 3 hr was applied to the duodenum by a trap under gastroscopy. After 8–12 h, the portable recorder and the suit were recovered and the data of the recorder was exported. Two experienced physicians blinded to each other's results were selected to read the imaging. Different results were finally discussed for agreements. All patients underwent gastroscopy and colonoscopy before or after MGCE examination. Patients with positive findings in the small bowel underwent DBE (Figure 1). Patients were hospitalized for 2–3 days and followed up for more than 2 weeks in order to estimate adverse events.

### 2.5. Main Outcome Measurements

The main outcome measurements are as follows: (1) gastric examination time; (2) small bowel transit time; (3) the completion rate of stomach examination; (4) the completion rate of small bowel examination (the capsule across the ileocecal valve during examination time); (5) the diagnostic yield (the rate of positive findings) and sensitivity of MGCE in both the stomach and small bowel examinations; and (6) the occurrence of adverse events.

### 2.6. Statistical Analysis

All the data were represented by mean and standard deviation. Pearson's chi-squared test was used for comparisons of subgroups. Fisher's test was accurately used for a value less than 5. In this study, the *p* value of a double-tailed case less than 0.05 presented statistically significant differences. All statistical analyses were achieved by IBM SPSS version 2.0.

## 3. Results

### 3.1. Study Populations

84 patients were finally enrolled in this study, with an average age of 12 ± 3.2 years, of which 53 (63.1%) were male. Subgroup I included 35 cases with abdominal pain (41.7%), and the levels of C-reactive protein (CRP) and erythrocyte sedimentation rate (ESR) were 18.6 ± 14.3 mg/l and 20.9 ± 12.7 mm/H, respectively. Subgroup II involved 22 cases with gastrointestinal bleeding: melena (6 patients, 7.1%), hematochezia (6 patients, 7.1%), hematemesis (3 patients, 3.6%), and anemia (7 patients, 8.3%). Patients with anemia had the average hemoglobin of 52.5 ± 14.0 g/l and underwent blood routine, blood smear, hemolytic anemia complete set, and bone wear, but there were no evidences of hematological diseases. They all received blood transfusion. Subgroup III enrolled 8 IBD cases: 6 cases with IBDU (6 patients, 7.1%) and 2 cases with suspected Crohn's disease (2 patients, 2.4%). Subgroup IV involved 3 cases with diarrhea (3.6%); subgroup V included 16 cases with other gastrointestinal discomforts: dyspepsia (12 patients, 14.3%), abdominal distension (2 patients, 2.4%), constipation (1 patient, 1.2%), and high level of CA199 (1 patient, 1.2%). All the patients successfully swallowed the capsule robot without mistaken aspiration. The gastric examination time was 12.1 ± 6.2 min. The mean small intestinal transit time was 248.5 ± 97.7 min. Two patients got metoclopramide to promote gastrointestinal dynamics. A patient's capsule was sent to the duodenum by gastroscopy. All patients completed gastric examination. A case (1.2%) did not complete the small bowel examination and the capsule reached the terminal ileum. During the examination, no patient felt discomfort. All capsules were discharged within two weeks, without capsule retention, intestinal obstruction, perforation, or mistaken aspiration.

### 3.2. Positive Findings

#### 3.2.1. Positive Findings in the Stomach

**Figure 2:**
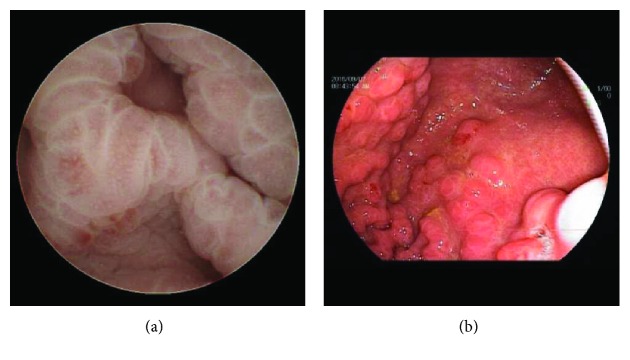
Positive findings in the stomach: (a) gastric mucosal nodular change with fold hypertrophy found by magnetic-guided capsule endoscopy; (b) gastric mucosal nodular change with fold hypertrophy verified by gastroscopy.

Eleven patients had lesions in their stomach (13.1%), including 2 patients (3.6%) with gastric inflammatory hyperplasia, 2 (2.4%) with exudative gastritis, 2 (2.4%) with polyps, 1 (1.2%) with erosive lesions (1, 1.2%), 1 (1.2%) with an ulcer, 1 (1.2%) with ectopic pancreas, and 1 (1.2%) with mucosal nodular changes with plica hypertrophy (Figures 2(a) and 2(b)). All lesions were confirmed by gastroscopy. The sensitivity of MGCE was 100%. The patient diagnosed with ectopic pancreas was followed up for 2 years using MGCE with no lesion progression seen.

#### 3.2.2. Positive Findings in the Small Bowel

**Figure 3:**
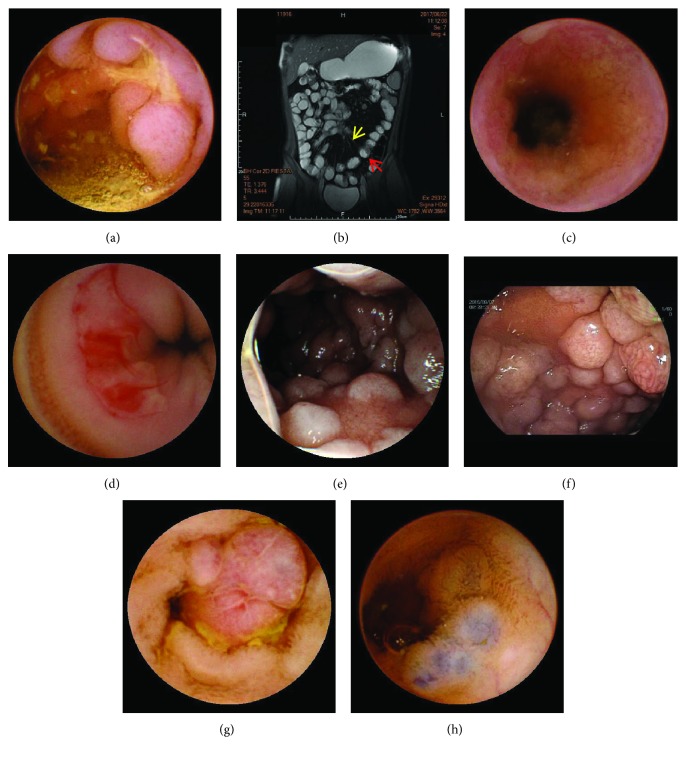
Lesions in the small bowel: (a) mucosal hyperplasia with ulceration found by magnetic-guided capsule endoscopy; (b) MRE plain scan, coronal plane, and FIESTA sequence; the red arrow showed the intestinal wall edema and the yellow arrow showed the vascular combs; (c) small bowel stenosis in CD seen by magnetic-guided capsule endoscopy; (e) small bowel ulcer with active bleeding showed by magnetic-guided capsule endoscopy; (f) mucosal segmental nodular change with nodular hypertrophy in the jejunum seen by magnetic-guided capsule endoscopy; (f) mucosal segmental nodular change with nodular hypertrophy in the jejunum verified by double-balloon endoscopy; (g) an adenomatous polyp found by magnetic-guided capsule endoscopy; (h) vascular malformations showed by magnetic-guided capsule endoscopy.

Of the 84 patients, 25 (29.8%) had positive findings on MGCE and all positive findings were confirmed by DBE.  Table 1 shows the positive findings on MGCE in the small intestine subgroups. Crohn's disease (CD) was the most common finding (10 patients, 11.9%) (Figures 3(a), 3(b), and 3(c)), and 9 patients were diagnosed by small bowel imaging including computed tomography enterography (CTE) and magnetic resonance enterography (MRE). All patients received systemic treatments and had symptom relief. Two patients underwent MGCE after 3 and 6 months of step-up treatments; one patient showed mucosal improvement on MGCE, and the other achieved complete mucosal healing. The second most common finding was Henoch-Schonlein purpura (4 patients, 4.8%) for which small bowel imaging was negative. Small intestinal ulcers (3 patients, 3.6%) (Figure 3(d)), intestinal polyposis (3 patients, 3.6%) (Figure 3(e)), vascular malformation (2 patients, 2.4%) (Figure 3(f)), intussusception (1 patient, 1.2%), active bleeding in the jejunum (1 patient, 1.2%), and non-Hodgkin's lymphoma confirmed by biopsy (Figures 3(g) and 3(h)) were also found on MGCE. In a patient in subgroup I, the capsule did not pass the ileocecal valve during the examining time, but there was no obvious abnormality found in the small intestine. This patient underwent colposcopy which showed no lesion in the terminal ileum. A patient with a negative MGCE was diagnosed with nonspecific enteritis by MRE. Other patients with a negative MGCE underwent other supplemental small intestinal imaging examinations including computed tomography (CT), CTE, MRE, and digital subtraction angiography (DSA). There were no missed diagnoses and no patient was misdiagnosed.

#### 3.2.3. Comparisons of Positive Findings on MGCE and CTE/MRE

The sensitivity of MGCE was significantly higher than that of CTE/MRE (96.0% vs. 52.0%, *p* = 0.001) (Table 2). When the purpura subgroup was compared, the sensitivity of MGCE was significantly higher than that of CTE/MRE (100% vs. 0%,*p* = 0.029). There was no significant difference in sensitivity in the CD subgroup (*p* = 0.50). Other positive findings could not be compared owing to the small sample size.

## 4. Discussion

The use of small bowel capsule endoscopy in pediatrics has increased since the US FDA extended the indication for capsule endoscopy (CE) to children over 2 years of age in 2009. Currently, in pediatrics, CE is mainly used for inflammatory bowel disease (IBD), obscure gastrointestinal bleeding (OGIB), anemia, abdominal pain, diarrhea, and polyposis [15]. However, in children less than 8 years of age, the incidence of IBD is low, and, as for adults, the main indication for CE is OGIB [14, 16, 17]. Because there is a risk of mistaken aspiration in children, the application of capsule endoscopy in pediatrics remains limited. During the last 10 years, we have accumulated some experience and skill in operating and reading MGCEs in minors, and we found that after scanning the stomach, the MGCE has enough battery power to provide high-quality images while investigating the small bowel in minors. Additionally, the diagnostic yield of MGCE is comparable to ordinary CE in the small intestine. Further, with MGCE, both gastric and small intestinal examination can be performed at the same time, which is well tolerated by minors. However, no studies focusing on this topic have been performed. In this study, with the collaboration of pediatricians, we aimed to investigate the safety, maneuverability, and clinical value of MGCE in the diagnosis of gastrointestinal diseases in minors.

In this study, the sensitivity of MGCE for gastric examination of minors was 100%, which is higher than that in adults (61.9%) [18]. The stomach cavity in a minor has a smaller volume and is softer than that in adults, making a minor's stomach easier to fill with water, which facilitates MGCE navigation. This may explain the high sensitivity found in minors.

In this study, MGCE had a high diagnostic yield of small bowel lesions in the IBD group, which is comparable to that of CE for the small intestine (87.5% vs. 86%) [16]. CE provides evidence for the identification of CD, the reclassification from ulcerative colitis/IBD unclassified to CD, and the adjustment of medical strategy [19, 20]. In addition, CE can predict long-term therapeutic effect and is a means for independent follow-up in CD patients [21]. Furthermore, the MGCE used in this study can help assess gastric involvement in pediatric patients. Therefore, CD could be considered the first indication for MGCE examination in minors.

Gastrointestinal bleeding is another important indication for MGCE. In group II, the diagnostic yield of MGCE is 31.8%. In an adult with OGIB, the diagnostic yield of CE ranges from 32% to 83% [22] and the major causes are vascular malformation, CD, and small bowel tumor [23], which are different from those in minors. In this study, more than 60% of minors with gastrointestinal bleeding had a negative MGCE. In adults, a negative CE provides evidence for a low risk of rebleeding [24]. However, the false negative rate of CE is approximately 19%, especially for the diagnosis of vascular malformation, Meckel's diverticulum, and small intestinal malignant tumor [25]. However, systematic studies focusing on the clinical value of CE in pediatric OGIB patients are limited [2, 5, 13, 26–29] and there are no related studies of MGCE. Therefore, the etiology of gastrointestinal bleeding in minors and the sensitivity, specificity, and predicting factors of MGCE require a study.

Until now, the indications and clinical value of CE in patients with abdominal pain were controversial. In this study, the main causes were Henoch-Schonlein purpura and CD, which are different from those in adults. The diagnostic yield of CE in adult patients with abdominal pain ranges from 20.9% to 24.4%, and the main cause is CD, followed by tumor [30–32]. In this study, the diagnostic yield (25.7%) was slightly higher than that of the adults. The reasons for this could be (1) the patients recruited were hospitalized, and the syndrome may be more severe in them in outpatients; (2) the patients enrolled in this study had elevated C-reactive protein or erythrocyte sedimentation rate, which may be an effective predictor for positive CE findings in patients with abdominal pain [33, 34]; and (3) minors involved in this study had a high incidence of Henoch-Schonlein purpura, which was not frequently found in adult patients. Therefore, large-scale clinical trials confirming the clinical value of MGCE in minors with abdominal pain are still needed.

In the diarrhea subgroup, one patient was diagnosed with CD, and the diagnostic yield of MGCE was higher than that of the adults using CE (33.3% vs. 14%) [35]. Although CE is not recommended as a first-line examination for diarrhea, it facilitates making the diagnosis in 9% of patients with negative conventional endoscopy and imaging examination results [35]. The most common cause is IBD, and hematochezia and hypoalbuminemia on CE are positive predictors for it [36]. There is a lack of clinical studies on CE for patients with dyspepsia, constipation, high level CA 19-9, and abdominal distention, especially in the pediatric population. MGCE may not be suitable for such patients as a first-line procedure, but it remains valuable for suspected IBD in minors.

Currently, CTE and MRE are widely used for diseases of the small intestine, and the diagnostic yield is comparable to that of CE [37, 38]. In this study, MGCE had a better sensitivity than that of CTE and MRE in minors. The possible reasons for this advantage maybe as follows: (1) MGCE is better for scanning the lesions on the mucosal surface, which is where lesions in minors primarily occur; and (2) minors have difficulty cooperating with the CTE and MRE examination processes. However, CTE and MRE can provide supplementary information and, for safety, are still recommended before MGCE in minors to avoid capsule retention.

Swallowing problems in minors during MGCE remains a challenge, especially for patients younger than 8 years of age. We thoroughly explained the procedure process and techniques to the patients and their guardians to comfort them and reduce fear. Patient guidance and encouragement from doctors can help children swallow the capsule smoothly, even for those who are 6 years of age [39]. The overall incidence rate of adverse events is 2% [40], and capsule retention is one of the most common complications, especially for CD patients and patients less than 10 years of age [41]. In children, the high risk of capsule retention may be due to the narrow diameter of the intestinal cavity and the high incidence of CD-related intestinal stenosis. However, during our follow-up period, no adverse events occurred. We consider MGCE to be a safe procedure for minors.

There are some limitations to this study. First, the efficiency of MGCEs in the subgroups could not be compared owing to the small sample size. Second, longer follow-up time is needed to confirm the cure rate, recurrence rate, and accuracy of the original diagnosis. Finally, due to potential swallowing problems, patients less than 6 years of age were not recruited for this study, which may have led to some bias.

## 5. Conclusion

MGCE is a safe, well-tolerated, and feasible procedure with a high diagnostic yield and sensitivity in minor patients. It has important clinical value and great prospects in the diagnosis of gastrointestinal diseases in minors.

## Figures and Tables

**Table 1:** Positive findings of MGCE in the small intestine for subgroups (*N* = 84). MGCE: magnetic-guided capsule endoscopy; IBD: inflammatory bowel disease; CD: Crohn's disease; DY: diagnostic yield, the rate of positive findings; VM: vascular malformation.

Subgroups (*N*, DY%)	Purpura	CD	Ulcer	Intussusception	VM	Lymphoma	Bleeding	Polyposis
(I) Abdominal pain (9/35, 25.7)	3	1	2	1	1	1	0	0
(II) Bleeding (7/22, 31.8)	1	1	1	0	1^#^	0	1	2
(III) IBD (7/8, 87.5)	0	7	0	0	0	0	0	0
(IV) Diarrhea (1/3, 33.3)	0	1	0	0	0	0	0	0
(V) Others (*n* = 1/16, 6.3)	0	0	0	0	0	0	0	1

^#^Blue rubber bleb nevus syndrome.

**Table 2:** Comparisons of positive findings on MGCE vs. CTE/MRE (*N* = 25). MGCE: magnetic-guided capsule endoscopy; CTE: computed tomography enterography; MRE: magnetic resonance enterography; VM: vascular malformation.

Positive findings	CE (*N*)	CTE/MRE (*N*)	Confirmed cases (*N*)	Miss rate (%)	
				CE	CTE/MRE
Purpura	4	0	4	0	100
CD	10	9	10	0	10
Ulcer	3	0	3	—	—
Intussusception	1	1	1	—	—
VM	2	1	2	—	—
Lymphoma	1	1	1	—	—
Bleeding	1	0	1	—	—
Polyposis	2	0	2	—	—
Enteritis	0	1	1	—	—

## Data Availability

All data can be acquired by connecting the corresponding authors.
